# Genome-based approach to evaluate the metabolic potentials and exopolysaccharides production of *Bacillus paralicheniformis* CamBx3 isolated from a Chilean hot spring

**DOI:** 10.3389/fmicb.2024.1377965

**Published:** 2024-04-02

**Authors:** Manik Prabhu Narsing Rao, Ram Nageena Singh, Rajesh K. Sani, Aparna Banerjee

**Affiliations:** ^1^Instituto de Ciencias Aplicadas, Facultad de Ingeniería, Universidad Autónoma de Chile, Sede Talca, Talca, Chile; ^2^Department of Chemical and Biological Engineering, South Dakota Mines, Rapid City, SD, United States; ^3^2-Dimensional Materials for Biofilm Engineering, Science and Technology, South Dakota Mines, Rapid City, SD, United States; ^4^Data Driven Material Discovery Center for Bioengineering Innovation, South Dakota Mines, Rapid City, SD, United States; ^5^BioWRAP (Bioplastics With Regenerative Agricultural Properties), Rapid City, SD, United States

**Keywords:** *Baciilus paralicheniformis* CamBx3, bioactive compound, exopolysaccharides, genome analysis, pangenome

## Abstract

In the present study, a thermophilic strain designated CamBx3 was isolated from the Campanario hot spring, Chile. Based on 16S rRNA gene sequence, phylogenomic, and average nucleotide identity analysis the strain CamBx3 was identified as *Bacillus paralicheniformis*. Genome analysis of *B. paralicheniformis* CamBx3 revealed the presence of genes related to heat tolerance, exopolysaccharides (EPS), dissimilatory nitrate reduction, and assimilatory sulfate reduction. The pangenome analysis of strain CamBx3 with eight *Bacillus* spp. resulted in 26,562 gene clusters, 7,002 shell genes, and 19,484 cloud genes. The EPS produced by *B. paralicheniformis* CamBx3 was extracted, partially purified, and evaluated for its functional activities. *B. paralicheniformis* CamBx3 EPS with concentration 5 mg mL^−1^ showed an optimum 92 mM ferrous equivalent FRAP activity, while the same concentration showed a maximum 91% of Fe^2+^ chelating activity. *B. paralicheniformis* CamBx3 EPS (0.2 mg mL^−1^) demonstrated *β*-glucosidase inhibition. The EPS formed a viscoelastic gel at 45°C with a maximum instantaneous viscosity of 315 Pa.s at acidic pH 5. The present study suggests that *B. paralicheniformis* CamBx3 could be a valuable resource for biopolymers and bioactive molecules for industrial applications.

## Introduction

Thermal environments are considered pinpoint anomalies against a background of ambient life, they contain the most profound insights into the earliest life on Earth (Walter, 1996) and it is assumed that life on Earth evolved in such an environment (Woese, 1987). Culture-dependent and independent microbial diversity analysis of the thermal environment showed that they have diverse microbial diversity and harbor novel candidates (Luo et al., 2020; Narsing Rao et al., 2020, 2021). Ever since the exploration of Taq DNA polymerase from *Thermus aquaticus* and its application in the development of the polymerase chain reaction (Chien et al., 1976; Brock, 1997), enzymes of thermophiles have been of considerable interest. Apart from thermostable enzymes, thermophiles are a valuable resource for biopolymers (Wang et al., 2019, 2020, 2021; Banerjee et al., 2022). Thermophilic bacteria produce a variety of macromolecules, including exopolysaccharides, as adaptations to help microbial communities withstand extreme temperatures (Wang et al., 2021). Exopolysaccharides from thermophiles have significant potential due to their thermostability and biological activities, which include biocompatibility, antioxidant properties, non-cytotoxicity, antiviral and immunostimulant effects (Arena et al., 2009; Wang et al., 2021). Exopolysaccharides (EPSs) from thermophiles maintain the viscosity of oil drilling fluids at high temperatures and thus can be considered flocculating agents (Dhagat and Jujjavarapu, 2021; Gongi et al., 2021).

Genome sequencing coupled with advancements in bioinformatic tools provides valuable insights into bacterial evolution, ecology, taxonomy, pathogenesis, metabolism, and the design of related therapeutic interventions (Goris et al., 2007; Donkor, 2013; Wang et al., 2022). Our earlier microbial analysis of Chilean hot springs showed that they harbor bacteria with thermostable EPSs and bioactive molecules (Banerjee et al., 2022; Marín-Sanhueza et al., 2022). In continuation of our earlier work in the screening of bioactive molecules and biopolymers from Chilean hot springs, a strain designated CamBx3 was isolated. Initial screening findings indicated that the strain CamBx3 generated EPS. This work aimed to identify genes linked to exopolysaccharide (EPS) production, metabolic capabilities, and heat stress response mechanisms in strain CamBx3 by analyzing its genome. We isolated and purified the EPS from strain CamBx3 to assess its structural characteristics and functional capabilities.

## Materials and methods

### Isolation of strain CamBx3 and preliminary screening of EPS

Strain CamBx3 was isolated by serial dilution method from the water sample (at a depth of 0.5 m) collected from the Campanario hot spring (35°56′23″ S 70°36′22″ W) located in the central Andean Mountain of Chilean Maule region using nutrient agar. The surface water temperature was 56.4°C, with a pH value of 5.8, hence the same incubation conditions were employed for isolation. Strain CamBx3 was subjected to Gram staining (Gram, 1884). The shape and size of the strain CamBx3 were observed using nucleic acid stain DAPI (excitation/emission = 359 nm/461 nm) using a Leica Stellaris 5 confocal microscope (Leica Microsystems, Wetzlar, Germany). The preliminary production of EPS was analysed using scanning electron microscopy (FESEM, JEOl-JSM 7610FPlus) by following the protocol described by Banerjee et al. (2024).

### EPS production and purification

EPS production, recovery, and partial purification were carried out as described by Rimada and Abraham (2003) with a little modification. Strain CamBx3 was grown in nutrient broth at 55°C, pH 5.8 for 3 days. Then, the stationary phase bacterial cells harvested in nutrient broth (NB) (Difco) were treated with 4% trichloroacetic acid (w/v) for 30 min at 37°C to remove the proteins. The cells were centrifuged at 4°C, 5000 × *g* for 20 min to precipitate the proteins out. An equal volume of chilled ethanol was added to the chilled cell-free supernatant and left overnight at 4°C. The solvent-coagulated EPS was then separated by centrifuging the solution at 4°C and 12,000 × *g* for 20 min. The protein-purified EPS was then dialyzed, followed by lyophilization to obtain the EPS powder to see the functional activity. Optimally, 1.75 g L^−1^ of EPS production was achieved.

### Functional activity of the EPS

#### Antioxidant activity of the EPS

To understand the antioxidant activity, the Ferric Reducing Antioxidant Power (FRAP) activity was determined according to the method of Benzie and Strain (1996) with some modifications using a FRAP assay kit (BioVision, Milpitas, United States). The reaction mixture consisted of 10 μL of sample EPS solution (0.2, 0.5, 1.0, 2.0, and 5.0 mg mL^−1^) with 152 μL of FRAP assay buffer, 19 μL of ferric chloride (FeCl_3_), and 19 μL of FRAP probe. The reaction mixture was further incubated at 30°C for 60 min in dark condition. The absorbance of the mixture was measured at 594 nm in Mobi-Microplate Spectrophotometer (μ2 MicroDigital, Seoul, South Korea). The FRAP activity was calculated using Eq. 1:


(1) $$\left(B\times \frac{D}{V}\right)$$

Where B is the amount of ferrous ammonium sulfate from the standard curve (nmol), D is the dilution factor, and V is the volume of sample added to the reaction well (in μL). For the calibration curve, different concentrations of the ferrous standard were provided in the kit.

The Fe^2+^ ion chelating activity was analyzed according to Shi et al. (2013). The reaction mixture was composed 50 μL of the sample. EPS (0.2, 0.5, 1.0, 2.0, and 5.0 mg mL^−1^), 2 μL of 2 mM ferrous chloride (FeCl_2_) solution, 10 μL of 5 mM ferrozine (Sigma) solution, and 138 μL of distilled water. The reaction mixture was incubated at 30°C for 10 min in dark condition. The absorbance of the mixture was measured at 562 nm in Mobi-Microplate Spectrophotometer (μ2 MicroDigital, Seoul, South Korea). The Fe^2+^ chelating activity was calculated using Eq. 2:


(2) $$\left(1-\frac{A_{1}-A_{2}}{A_{0}}\right)\times 100$$

Where A_0_ is the absorbance of the control (water instead of the sample EPS solution), A_1_ is the absorbance of the EPS samples, and A_2_ is the absorbance with the EPS sample but without the ferrozine solution. EDTA was used as the positive control along with commercial bacterial EPS xanthan gum (Sigma).

#### Glucosidase inhibition activity

*β*-glucosidase (*β*-GA) inhibition activity of the EPS was performed using *β*-GA assay kit (Sigma-Aldrich). For this, 200 μL assay buffer and 8 μL of *β*-NPG substrate was mixed to reach a final concentration of 1 mM *β*-NPG. From this, 20 μL of the mix was added to 200 μL of EPS samples (0.2, 0.5, 1.0, 2.0, 5.0 mg mL^−1^) diluted in 50 mM phosphate buffer. About 200 μL of the reaction mixture was added to each well. The initial absorbance was measured at 405 nm with subsequent incubation at 30°C for 20 min. The final absorbance was taken again at 405 nm in Mobi-Microplate Spectrophotometer (μ2 MicroDigital, Seoul, South Korea) using the end-point technique. Eq. 3 was followed to determine the *β*-GA inhibition activity:


(3) $$\beta -GA=\frac{\left({\mathrm{Abs}}_{405}\right)\;\mathrm{final}-\left({\mathrm{Abs}}_{405}\right)\;\mathrm{initial}}{\left({\mathrm{Abs}}_{405}\right)\;\mathrm{calibrator}-\left({\mathrm{Abs}}_{405}\right)\;\mathrm{water}}\times 250\;\left(\frac{\mathrm{units}}{l}\right)$$

#### Rheological property analysis

To analyze the rheological properties of the EPS, 2% hydrocolloidal, aqueous EPS suspension was prepared at 30°C under continuous stirring. Hysteresis plots were recorded by varying the pH level (pH 3, 5, 7, and 9), and temperature (35°C and 45°C) using a dynamic rheometer (MCR 52, Anton Paar, Austria) by execution of small amplitude oscillatory shears (Banerjee et al., 2020). Temperature sweep experiment was performed for 2% EPS suspension at varying pH. For this experiment, evaporative losses were minimized by covering the exposed sample edges with a thin layer of low-viscosity mineral oil. Heating (40–90°C) and successive cooling (90–40°C) was done at 1.5°C min^−1^ and 1 Hz frequency, followed by establishing 0.6 Pa constant stress.

### Genome sequencing and analysis

Genomic DNA from strain CamBx3 was extracted using the ZymoBiomics DNA/RNA miniprep kit (Zymo Research) according to the manufacturer’s instructions. DNA concentration and quality (OD_260/280_ = 1.8) was assessed by using Nanodrop 1,000 (Thermo Fisher Scientific) and Qubit 4.0 (Thermo Fisher Scientific). The genomic DNA was also checked on 0.7% agarose gel for high molecular size along with Lambda DNA HindIII cut DNA marker (NEB, Inc.). The genomic DNA was sequenced using Oxford-Nanopore single molecule real-time sequencing technology (ONT-SMRT) at Meta-Omics Lab facility of South Dakota Mines (SDM), Rapid City, SD, USA. High-quality genomic DNA was processed for library preparation using a ligation sequencing kit (SQK-LSK109), as recommended (DNA damage repair and end-repair/dA tailing, adapter ligation) by Oxford-Nanopore. The sequencing library was purified three times with AMPureXP beads (Beckman Coulter Genomics, MA, United States) as per the oxford-nanopore library preparation recommendation for purification and capturing of desired library fragment size (∼10 kb) for sequencing. The final library was quantified on Qubit 4.0 and 100 fmol of DNA library was loaded with sequencing loading beads in a flow cell (R9.4.1) by following standard procedures. The flow cell was placed on MinION sequencer and sequencing was achieved by 12 h sequencing run with MinKNOW (v22.03.6) with super accuracy base call option (Guppy v6.0.7) (Wick et al., 2019). The quality passed raw sequence data generated from Nanopore sequencing was further processed for the assembly of reads to generate a chromosome-level genome assembly. The super high quality 558,043 reads (241,090,1974 bp) with *N*_50_ 10.92 kb were assembled using flye (v2.8.3) (Kolmogorov et al., 2020) with 3 iterations of Minimap2 v2.24 (Li, 2018).

The assembled genome has coverage of 529×. After the genome assembly, a separate polishing step was performed using Medaka (v1.6.0) with default parameters.^1^ The final polished and finished genome assembly was processed for characterization and annotation. The genome sequence of strain CamBx3 was submitted to GenBank under the accession number GCA_026210435.

The genome quality was estimated by CheckM (Parks et al., 2015). The genome was visualized by using Proksee (Seemann, 2014; Grant et al., 2023). The rRNAs and tRNAs were predicted using RNAmmer (Lagesen et al., 2007) and tRNAscan-SE, respectively (Lowe and Eddy, 1997). The 16S rRNA gene obtained from the CamBx3 genome was compared with the sequences in the NCBI GenBank database and EzBioCloud server (Yoon et al., 2017). The phylogenomic tree was reconstructed using the Anvi’o platform (Eren et al., 2015). The genes in HMM source “Bacteria_71” (Lee, 2019) were taken and aligned using MUSCLE (Edgar, 2004). The resulting tree was visualized using MEGA version 7.0 (Kumar et al., 2016). The average nucleotide identity (ANI) value was calculated using pyani software package with the ANIb parameter (Pritchard et al., 2016).

Functional annotation was performed using Anvi’o version 7.1 platform (Eren et al., 2015). The fasta file was first reformatted with anvi-script-reformat-fasta. The scripts anvi-gen-contigs-database and anvi-run-hmms were executed; this command uses Prodigal to identify open reading frames (Hyatt et al., 2010). Functional annotation was performed by KofamKOALA (Aramaki et al., 2020) using the anvi-run-kegg-kofams program. Pan-genome analysis was carried out using Roary (Page et al., 2015) using genome annotation data (GFF3 format) generated by Prokka (Seemann, 2014). The coding regions were extracted from the input and converted to protein sequences, filtered to remove partial sequences, and iteratively pre-clustered with CD-HIT (Fu et al., 2012), then an all-against-all comparison was performed with a built-in BLASTP on the reduced sequences with the default sequence identity cutoff. The Roary’s gene presence/absence dataset was used to build a Venn diagram.

### Statistical analysis

All the experiments were performed in triplicate; the statistical analysis was carried out using ANOVA.

## Results and discussion

### Identification of strain CamBx3 and preliminary screening of EPS

**Figure 1:**
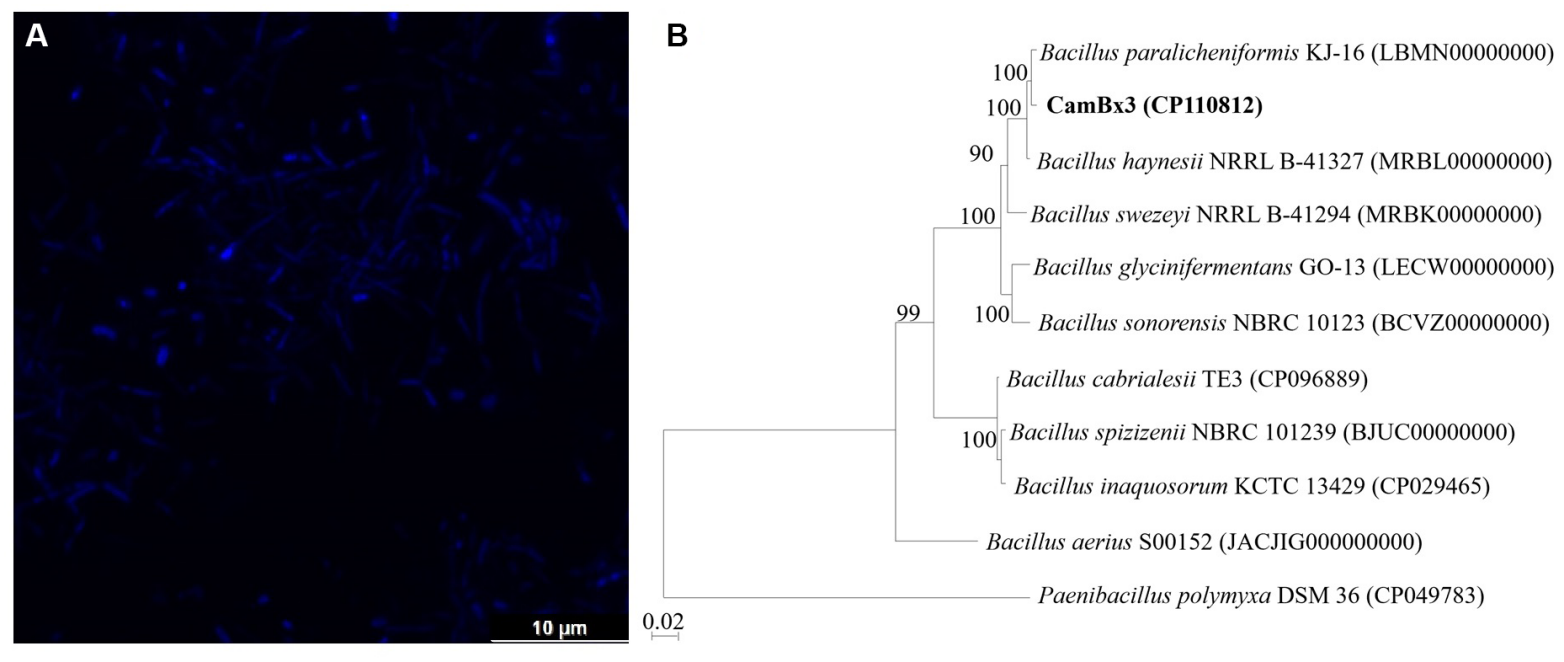
**(A)** Confocal micrograph of *B. paralicheniformis* CamBx3 showing the presence of rod-shaped cells; **(B)** Phylogenomic tree based on 16S rRNA gene sequence showing the relationships of *B. paralicheniformis* CamBx3. Bootstrap values (expressed as percentages of 1,000 replications) greater than 50% are shown at branch points. Bar, 0.02 substitutions per nucleotide position.

Strain CamBx3 was Gram-stain-positive and creamish white in color. The confocal micrograph revealed that the strain CamBx3 exhibited a rod-shaped morphology (Figure 1A). The 16S rRNA gene sequence extracted from CamBx3 genome showed the highest similarity with *Bacillus paralicheniformis* (100%). In the phylogenomic tree, CamBx3 clade with *B. paralicheniformis* (Figure 1B). The above results suggest that strain CamBx3 was a member of the genus *Bacillus*. Additionally, it showed a mucoid colony appearance indicating EPS production. The SEM analysis (Supplementary Figure S1) revealed the presence of EPS and optimally, 1.75 g L^−1^ of EPS production was achieved.

### Genome attributes of strain CamBx3

**Figure 2:**
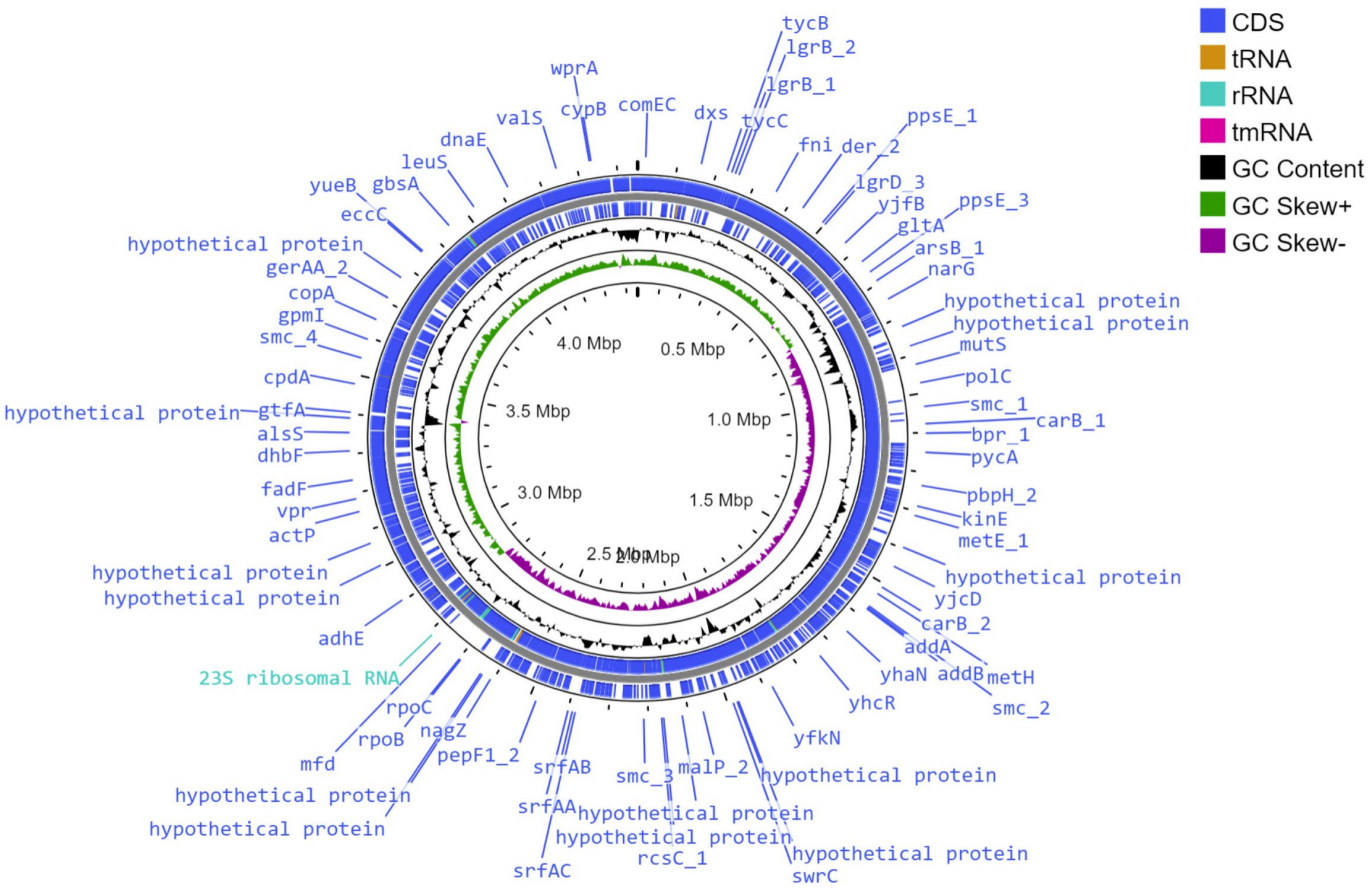
Circular genome view of *B. paralicheniformis* CamBx3 created using Proksee.

The whole genome sequence of strain CamBx3 was assembled in a single circular chromosome (Figure 2) and contains 4,452,754 bp (4.45 Mb). The genomic DNA G + C content was 45.8% and the genome completeness and contamination were 99.5 and 0.1%, respectively, indicating a high-quality genome (Parks et al., 2015). A total of 4,648 genes including 4,541 protein-coding genes, 83 tRNAs and 24 rRNAs were predicted. The ANI value (Supplementary Table S1) of CamBx3 was highest with *B. paralicheniformis* KJ-16 (96.9%). The ANI value between CamBx3 and *B. paralicheniformis* KJ-16 (96.9%) was above the cut-off level (95–96%) for species delineation (Richter and Rosselló-Móra, 2009). The above results suggest that CamBx3 and *B. paralicheniformis* were similar species.

### Genes related to EPS

Generally, the biosynthetic process of EPSs comprises regulation, chain-length determination, repeat-unit assembly, polymerization, and export (Ates, 2015). EPS biosynthetic pathway involves the sugar uptake system, and the most efficient sugar transport is the phosphoenolpyruvate-phosphotransferase system (PEP-PTS) (Xiong et al., 2019). The genes responsible for glucose, glucosamine, *β*-glucoside, *N*-acetylglucosamine, fructose, mannose, oligo-*β*-mannoside, cellobiose, maltose, trehalose, mannitol, ascorbate, glucitol/sorbitol, and oligo-*β*-mannoside-specific PEP-PTS systems were noticed in *B. paralicheniformis* CamBx3 genome. Sugars that do not have a particular PEP-PTS were delivered into the cytoplasm via non-PEP-PTS systems (Cui et al., 2016). The genes related to the L-arabinose transport system permease protein, lactose transport system permease protein, D-xylose transporter, and sucrose permease were also identified in the strain CamBx3 genome. These results suggest that strain CamBx3 may uptake glucose, fructose, sucrose, maltose, mannose, galactose, xylose, lactose, arabinose, and cellobiose.

**Figure 3:**
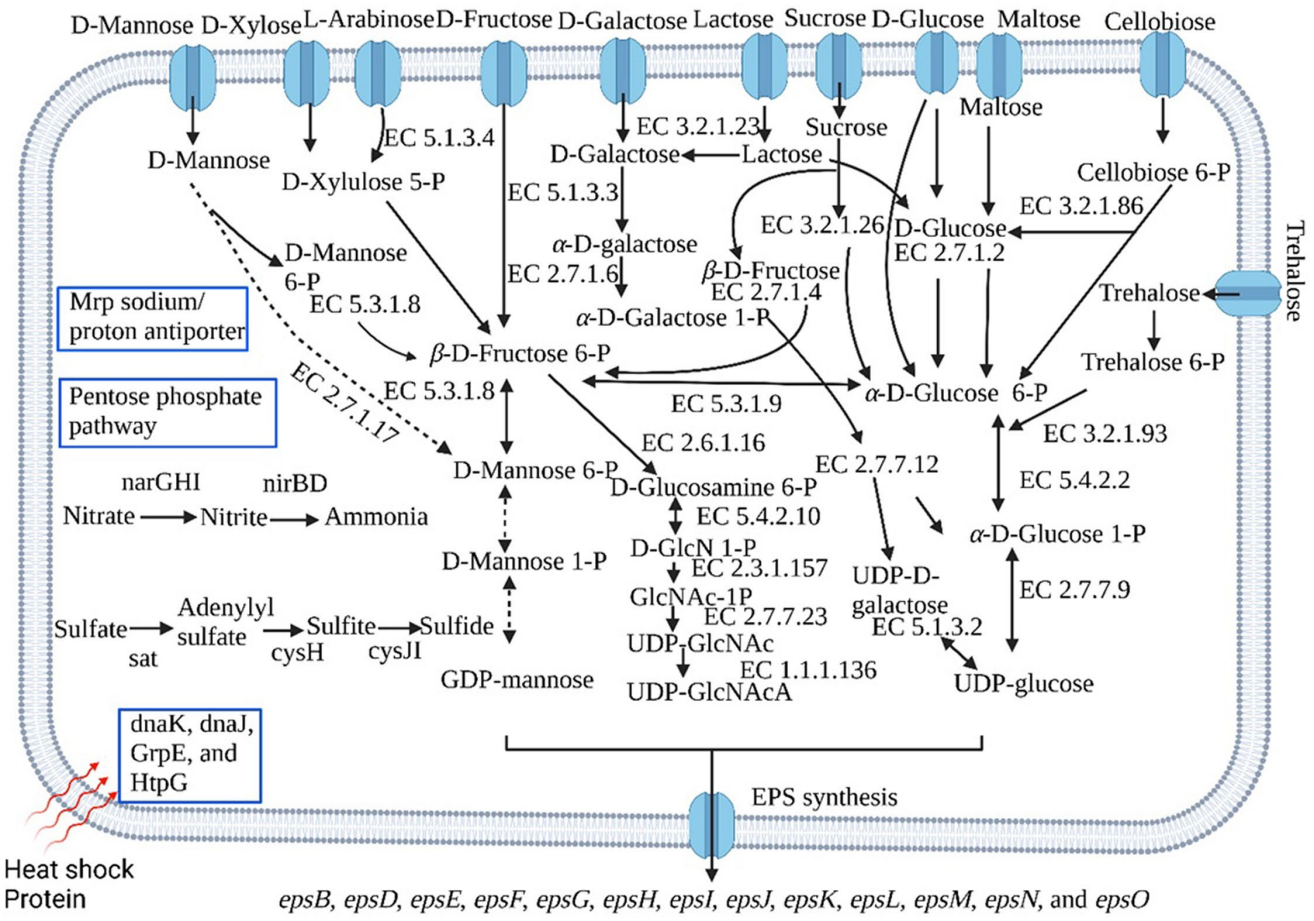
Some important metabolic pathways of *B. paralicheniformis* CamBx3 based on *KEGG* (Kyoto Encyclopedia of Genes and Genomes) *databases*. The dashed line arrow indicates the absence of the gene. The figure was created by BioRender.com.

Once sugars enter the cytoplasm, they will be converted to nucleotide sugars via different pathways (Figure 3), which act as active precursors in synthesising the EPS structure. The glycolysis pathway is the first stage in EPS production, and the key enzymes for EPS production include UTP-glucose-1-phosphate uridylyltransferase and uridine diphosphate (UDP)-glucose-4-epimerase for UDP-glucose biosynthesis, and mannose-1-phosphate guanylyltransferase for GDP-mannose biosynthesis (Wang et al., 2019). Glucose is phosphorylated by glucokinase to glucose-6-phosphate, then mutated to glucose-1-phosphate by phosphoglucomutase. UTP-glucose-1-phosphate uridylyltransferase might convert glucose-1-phosphate to UDP-glucose (Li et al., 2018). The enzymes for the conversion of glucose to UDP-glucose were noticed in *B. paralicheniformis* CamBx3 (Figure 3). CamBx3 also contained enzymes for the breakdown of lactose into glucose and galactose (through *β*-galactosidase) and the conversion of galactose to glucose-1-phosphate to UDP-galactose and UDP-glucose. Sucrose, mannose, xylose, and arabinose were catalyzed to fructose-6-phosphate and then converted to UDP-*N*-acetyl-*α*-D-glucosamine and UDP-N-acetyl-2-amino-2-deoxy-D-glucuronate. The enzyme mannose-1-phosphate guanylyltransferase for GDP-mannose biosynthesis was not present in *B. paralicheniformis* CamBx3. These results indicate that *B. paralicheniformis* CamBx3 may use UDP-glucose, UDP-galactose, UDP-*N*-acetyl-*α*-D-glucosamine, and UDP-*N*-acetyl-2-amino-2-deoxy-D-glucuronate as primary precursors for the production of EPS. Recently, *Bacillus* sp. ISTL8 was reported for EPS production and uses UDP-galactose, dTDP-rhamnose, and UDP-glucose as primary precursors for the production of EPS (Gupta et al., 2021).

The genome analysis of *B. paralicheniformis* CamBx3 revealed the presence of EPS biosynthetic gene cluster (*epsB*, *epsD*, *epsE*, *epsF*, *epsG*, *epsH*, *epsI*, *epsJ*, *epsK*, *epsL*, *epsM*, *epsN*, and *epsO*). The EPS gene cluster might assemble the nucleotide sugars listed above into repeating units to create EPS. Genes *epsA* and *espB* regulate EPS biosynthesis (Xiong et al., 2019). EPS chain length is determined by the genes *epsC* and *epsD*, while biosynthesis of repeating sugar units is encoded by *epsE*, *epsF*, *epsG*, *epsH*, and *epsI*, and EPS polymerization and export are encoded by *epsJ*, *epsK*, *epsL*, and *epsM* (Wu et al., 2020).

### Functional activity of the EPS

#### Antioxidant activity

**Figure 4:**
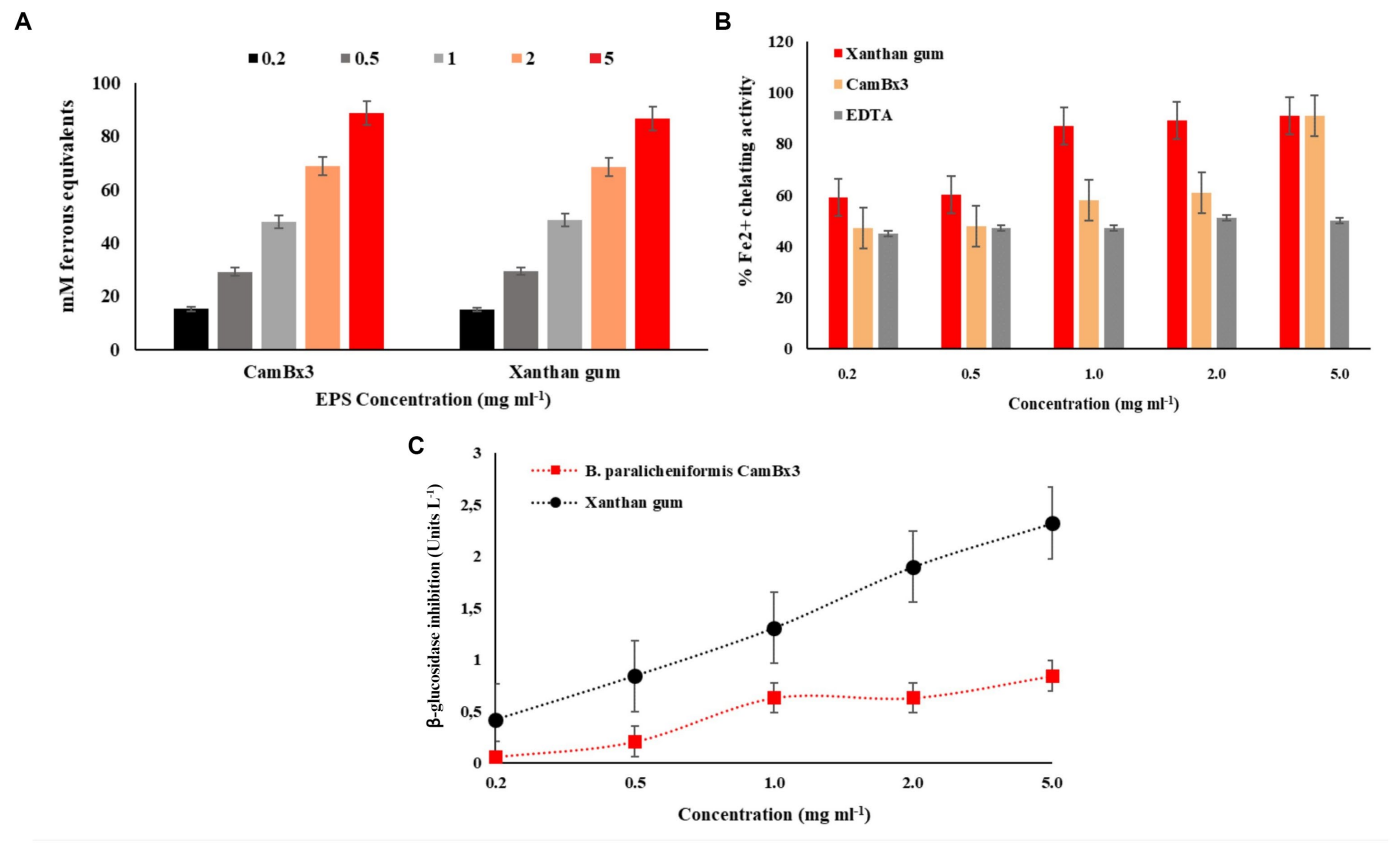
Antioxidant activity of the exopolysaccharide (EPS) produced by *B. paralicheniformis* CamBx3; **(A)** FRAP activity, and **(B)** Fe^2+^ chelating activity. **(C)** β-glucosidase enzyme inhibition activity.

In FRAP activity test, CamBx3 EPS demonstrated almost similar ferric-reducing activity to commercial bacterial EPS (xanthan) with more than 90 mM ferrous equivalents (Figure 4A). The ferrous equivalent was calculated by preparing the ferrous standard curve. The EPS produced by CamBx3 showed 92 mM ferrous equivalents (5 mg mL^−1^ of EPS), which was more than that of commercial bacterial biopolymer xanthan gum at the same concentration (Figure 4B). An exponential increase in the ferrous ion chelation was observed with increasing EPS concentration compared to the positive control.

Reactive oxygen species (ROS) are natural by-products of normal aerobic metabolism or host defence mechanisms involved in various biological processes (Juan et al., 2021). FRAP assay is a simple method to determine the antioxidant activity of a compound that reduces Fe^3+^ to Fe^2+^. The reducing potentials of antioxidants are associated with their electron-donating abilities to break the free radical chain reactions (Benzie and Strain, 1996). A very high FRAP value was recorded in our study similar to xanthan. The results indicated that the EPS might act as electron donors to react with free radicals and convert them into stable products terminating the free radical chain reactions. *In vitro* antioxidant activity supports the application of CamBx3 EPS for food and pharmaceutical applications as a natural antioxidant and product shelf-life enhancer.

With the increase in consumer-oriented functional food, natural antioxidants have received great attention from researchers because of their ability to inhibit ROS and radicals (Zhao and Liang, 2022). The Fe^2+^ chelating activity is considered an important antioxidant property. The transition of Fe^2+^ is reported to stimulate lipid peroxidation by generating hydroxyl radicals through the Fenton reaction via decomposing lipid hydroperoxides into peroxyl and alkoxyl radicals (Benedet and Shibamoto, 2008). Chelating agents inhibit lipid oxidation by stabilizing transition metals. In our study, the EPS produced by CamBx3 showed a maximum 91% Fe^2+^ ion chelation at a low concentration of EPS (5 mg mL^−1^) usage same as commercial bacterial polysaccharide xanthan. In a study, *Lactobacillus helveticus* MB2-1 reported to exhibit a chelating capacity on Fe^2+^ at 4.0 mg mL^−1^ of up to 59.1% (Li et al., 2014). More than 90% of *in vitro* Fe^2+^ chelation has been reported earlier for the EPS produced by endophytic *Paenibacillus polymyxa* EJS-3 (Liu et al., 2010), confirming the capacity of bacterial EPS to chelate Fe ions thereby possibly inhibiting the lipid peroxidation. The presence of –OH and –O– groups in structures of bacterial polysaccharides might be responsible for this property (Liu et al., 2010).

#### Glucosidase inhibition activity

*β*-glucosidase inhibitors are being extensively studied these days for their use as anti-diabetics, anti-obesity, and anti-tumor compounds (Chaipoot et al., 2023). So far, these compounds have been reported in large numbers from plants, algae, fungi, and marine bacteria (Pandey et al., 2013). The EPS produced by *B. paralicheniformis* CamBx3 showed *β*-GA inhibition activity even at a low concentration (0.5 mg mL^−1^). An increase in the inhibition was observed with increasing concentrations of EPS and commercial xanthan as a control in the studied concentrations (Figure 4C). For all bioactive compounds; there is a dose-response activity which results in the evolution of the percentage of inhibition according to the increase of concentrations (Chokki et al., 2020). Recent studies reported bioactive compounds from *Momordica charantia* Linn. leaves inhibited the activity of *β*-GA enzyme useful as antiviral, antiadhesive, antibacterial, antimetastatic, or immunostimulatory agents (Chokki et al., 2020). Human immunodeficiency virus (HIV), the causative agent of AIDS, contains two heavily glycosylated envelope proteins, gp120 and gp41, and it has been reported that an interaction between glycoprotein gp120 and the cellular protein CD4 is required to initiate the infection cycle (Hart et al., 1991). Metabolites capable of inhibiting *β*-glucosidase activity have shown anti-HIV activity by inhibiting glycoprotein processing, which in turn affects the formation of the syncytium and results in an alternative site of action for HIV (Gruters et al., 1987). Thus, our study demonstrates important bioactive potential of the CamBx3 EPS as *β*-GA inhibitor, which might have future applications in biomedicine or biopharmaceutical industry.

#### Rheological property of the EPS

**Figure 5:**
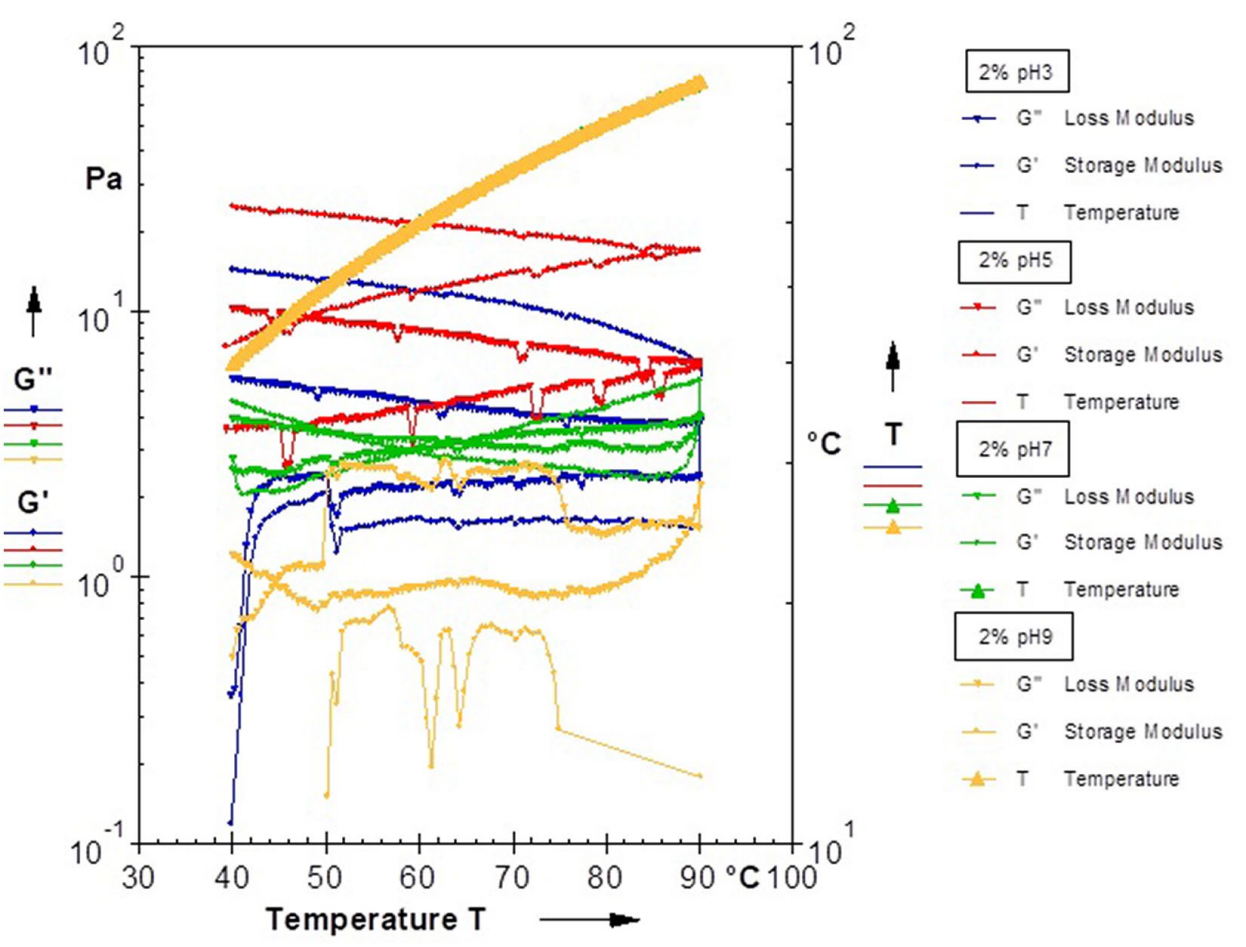
Rheological analysis showing temperature sweep of 2% aqueous EPS solution at different pH.

Exopolysaccharides have unique rheological and physicochemical characteristics and offer advanced functionality (Bamigbade et al., 2023). *B. paralicheniformis* CamBx3 EPS hydrocolloid at 35°C, compared to basic pH of 9.0, both at acidic and neutral pH has demonstrated formation of a gel resistant to flow (Figure 5). At low shear rate, the maximum instantaneous viscosity of 304 Pa.s was observed for a neutral EPS gel followed by a highly acidic EPS gel with 280 Pa.s at a pH of 3.0 (Table 1). An increase in temperature to 45°C resulted in the production of a viscoelastic acid gel with a maximal instantaneous viscosity of 315 Pa.s at pH 5.0 and 203 Pa.s at pH 3.0. Interestingly, the EPS gel at basic pH at both temperatures did show a low viscosity with poor shear thinning behavior. In fact, at 45°C, structural degradation of EPS was observed at pH 9.0 (Table 1). Improved viscoelasticity is typically associated with the structural reversibility of gels, which is a result of the formation of 3D networks facilitated by hydrogen bonding between polysaccharide macromolecules (Li et al., 2011). Acid gels have been noted for their use in drug delivery systems, particularly in seaweed polysaccharides (Zhong et al., 2020). In our study, a similar viscoelastic acid gel formation was observed at both 35°C and 45°C, with steady shear thinning behavior and structural reversibility, suggesting possible applications in the biopharmaceutical industry in the future. The temperature sweep curve of acid gel at pH 3.0 showed a characteristic stability from 50–90°C region followed by the neutral pH EPS gel at pH 7.0. The temperature sweep investigation, like the Carreau model hysteresis experiment, found that EPS has good viscosifying and rheological properties at pH 3.0 and 7.0, as well as structural degradation or instability at pH 9.0. The rheological properties of a polysaccharide are crucial for its application in the food business. Commercial bacterial EPS xanthan maintains structural integrity when it cools, indicating renaturation. In contrast, curdlan polysaccharide, typically insoluble in water, only gels when heated. This process is irreversible (Maalej et al., 2016). In our study, the gel-like behavior of CamBx3 was observed to be maintained even at high temperatures and cooling was favorable for the enhancement of a gel-like network probably due to the aggregation of the polymer chains at both acidic pH and neutral pH as reported earlier by Maalej et al. (2016).

**Table 1:** Dynamic rheological properties of the EPS at different temperatures under pH variation in terms of the Carreau model.

Temperature (°C)	pH	*η*_o_	*η*_∞_ (×10^−9^)	*A*	*p*-value	*R* ^2^	SD
35	3.0	280.26	3.8883e^-9^	96.60	0.49	0.999	0.19
	5.0	148.41	4.2631e^-9^	38.64	0.49	0.998	0.39
	7.0	303.80	3.8114e^-9^	82.47	0.50	0.999	0.39
	9.0	91.72	3.4499e^-9^	22.94	0.51	0.997	0.33
45	3.0	203.36	4.221e^-9^	58.97	0.49	0.998	0.65
	5.0	315.04	3.5481e^-9^	80.71	0.51	0.999	0.62
	7.0	177.02	3.6376e^-9^	42.77	0.51	0.994	0.96
	9.0	−15.62	–	–	–	–	–

Where *η*_o_, instantaneous viscosity; 𝜂∞, final viscosity after prolonged shearing; *A*, coefficient; *p*, measure of the shape of the curve between shear rate and shear stress; *R*, coefficient of correlation; SD, standard deviation.

### Genes related to nitrogen and sulphur metabolism

Nitrate is the most oxidized form of fixed nitrogen compounds and one of the most important nutrients for microbial and plant life (Kamp et al., 2015). In prokaryotes, dissimilatory nitrate reduction mechanisms have been extensively explored (Kamp et al., 2015; Sun et al., 2018; Keren et al., 2020). Dissimilatory nitrate reduction can occur in several ways, beginning with nitrate reduction to nitrite by respiratory membrane-bound *NarG* or periplasmic nitrate reductase *NapA*, followed by nitrite reduction to ammonia via cytoplasmic nitrite reductase *NirB* or periplasmic nitrite reductase *NrfA* (Sun et al., 2018). *Bacillus subtilis* anaerobically reduces nitrate to ammonium by nitrate reductase *NarGHI* and nitrite reductase *NirBD*, whereas *Bacillus selenitireducens* generates ammonium via the periplasmic nitrite reductase *NrfA* (Nakano et al., 1998; Nakano and Zuber, 1998). *B*. *paralicheniformis* CamBx3 reduces nitrate to ammonium via nitrate reductase (*NarGHI*) and nitrite reductase (*NirBD*) (Figure 3). Microorganisms use assimilatory sulfate reduction (ASR) pathway to convert inorganic sulfate to sulfide (Koprivova et al., 2001). The genes (*sat*, *cysH* and *cysJI*) involved in the ASR were identified in *B. paralicheniformis* CamBx3 (Figure 3). ASR pathway leads to the biosynthesis of sulfur-containing amino acids, such as cysteine, and does not lead to the direct excretion of sulfide (Kopriva et al., 2007; Longo et al., 2016).

### Stress-related genes

Microbes at high temperatures must maintain their protein machinery stable and efficient (Narsing Rao et al., 2022). *DnaK*, *DnaJ*, and *GrpE* from cellular chaperone machinery capable of repairing heat-induced protein damage (Schröder et al., 1993). *B. paralicheniformis* CamBx3 encodes genes for *Dnak*, *DnaJ*, *GrpE*, and *HtpG* (Figure 3). Multiple resistance and pH adaptation (Mrp) antiporters are multi-subunit complexes that link Na^+^ (or K^+^) ion transport across the membrane to the proton motive force. They play various physiological roles, including pH and Na^+^ homeostasis, as well as Na^+^ tolerance (Haja and Adams, 2021). *B. paralicheniformis* CamBx3 also encodes genes for Mrp sodium/proton antiporter. Ancient Mrp antiporter has been reported earlier in diverse thermophilic or hyperthermophilic, Gram-positive and Gram-negative bacteria along with archaea for adaptation in polyextremophilic environments (Ito et al., 2017).

### Carbohydrates, amino acids, and other metabolic pathways

Genes related to the pentose phosphate and Entner-Doudoroff pathways were present in *B. paralicheniformis* CamBx3. The shikimate pathway is the central metabolic route leading to the formation of tryptophan, tyrosine, and phenylalanine (Averesch and Krömer, 2018). The genes related to the shikimate pathway were noticed in *B. paralicheniformis* CamBx3 (Supplementary Table S2). Further, genes related to tryptophan biosynthesis were also noticed in strain CamBx3 (Supplementary Table S2). A detailed list of the metabolic potentials of *B. paralicheniformis* CamBx3 is mentioned in Supplementary Table S2.

### Pangenome analysis

**Figure 6:**
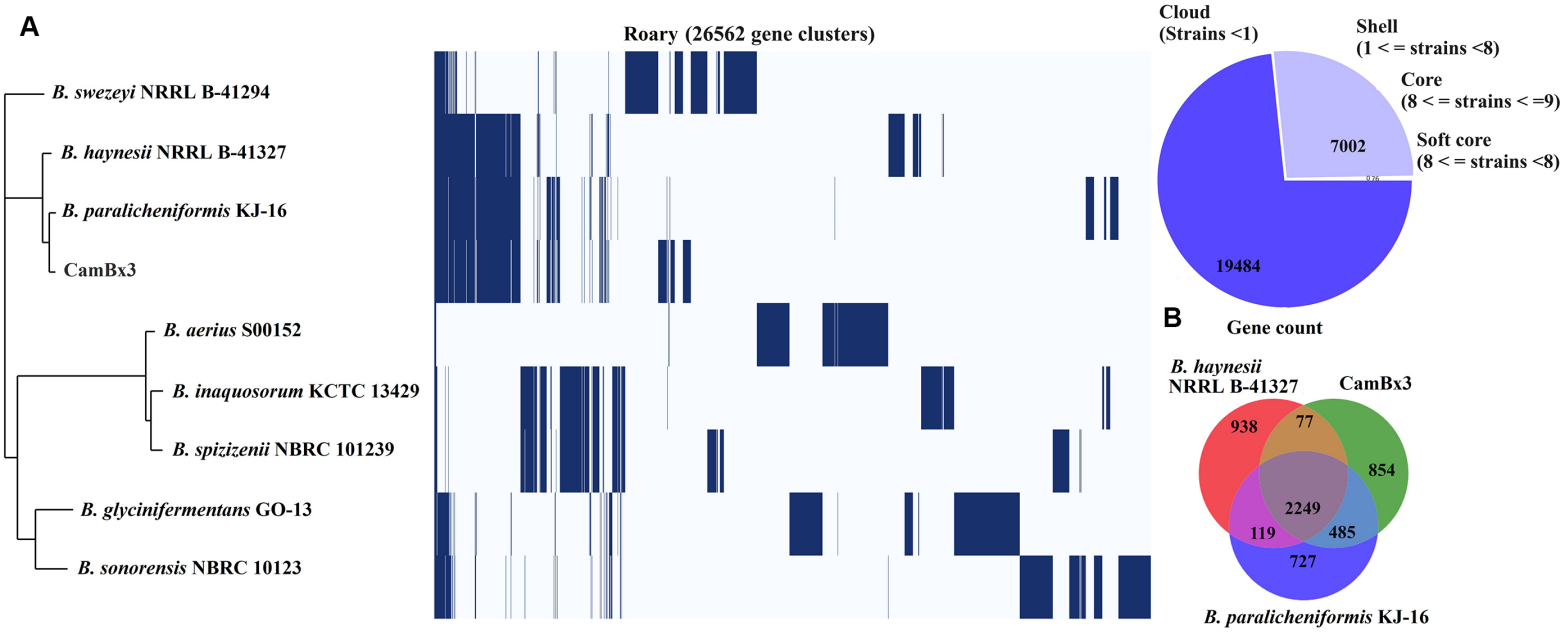
Pangenome analysis: **(A)** Roary gene cluster, showing the presence and absence of genes, **(B)** Venn diagram (based on presence and absence of genes) between *B. paralicheniformis* CamBx3 and two closely related *Bacillus* species.

The pangenome analysis of *B. paralicheniformis* CamBx3 was performed with eight *Bacillus* spp. Figure 6A, shows the Roary matrix, which includes 26,562 gene clusters generated from all 9 genomes. These gene clusters were categorized as 7,002 shell genes, and 19,484 cloud genes. Although strains *B. paralicheniformis* CamBx3 and *B. paralicheniformis* KJ-16 were similar species based on 16S rRNA, a clear difference was observed in their gene clusters shown in Figure 6A. A Venn diagram (using the presence and absence gene matrix) was constructed between *B. paralicheniformis* CamBx3, *B. paralicheniformis* KJ-16 and *B. haynesii* NRRL B-41327 (Figure 6B). A total of 854 different genes were found in *B. paralicheniformis* CamBx3 when compared to *B. paralicheniformis* KJ-16 and *B. haynesii* NRRL B-41327 (Supplementary Table S3). The above results show the differences in the genome content of *B. paralicheniformis* CamBx3. Comparing the gene clusters of CamBx3, NRRL B-41327, and KJ-16, EPS formation-related genes (*epsH*, *epsM*, and *epsF*), associated with glycosyl transferase experession, putative acyltransferase, and biofilm formation were noticed in three strains. Additionally, heat stress-related chaperones *dnaJ*, and *dnaK* were also encountered.

## Conclusion

In the present study, a thermophilic strain of *B. paralicheniformis*, designated CamBx3 was isolated from the water sample of Campanario hot spring located in Andean Mountain of central Chile. Genome analysis identified genes related to EPS clusters, assimilatory sulfate reduction, heat stress-related machinery along with the major metabolic potentials. Additionally, the pangenome analyses showed that *B. paralicheniformis* CamBx3 has very different genome content compared to the nearby species *B. paralicheniformis* KJ-16 and *B. haynesii* NRRL B-41327. The EPS produced by this thermophilic strain has demonstrated FRAP-mediated antioxidant capacity and Fe^2+^ ion chelating properties. The EPS is additionally a *β*-glucosidase enzyme inhibitor and rheologically formed a thermo-resistant, viscoelastic gel at acidic pH. Acid gels are known for their application in drug delivery systems. The EPS extracted from *B. paralicheniformis* CamBx3 showed strong antioxidant, and glucosidase inhibition activities, as well as suitable viscoelastic acid gel formation, which could be a valuable resource for biotechnological applications.

## Data availability statement

The datasets presented in this study can be found in online repositories. The names of the repository/repositories and accession number(s) can be found at: https://www.ncbi.nlm.nih.gov/, GCA_026210435.

## Author contributions

MN: Writing – review & editing, Writing – original draft, Software, Methodology, Investigation, Formal analysis. RSi: Writing – review & editing, Software, Methodology, Investigation, Formal analysis, Data curation. RSa: Writing – review & editing, Resources, Investigation. AB: Writing – review & editing, Writing – original draft, Supervision, Resources, Project administration, Methodology, Investigation, Funding acquisition, Formal analysis, Conceptualization.

## Footnotes

^1^ https://github.com/nanoporetech/medaka
