# Supplementary material for: Genome-based approach to evaluate the metabolic potentials and exopolysaccharides production of Bacillus paralicheniformis CamBx3 isolated from a Chilean hot spring
Source: Front Microbiol. 2024 Apr 2;15:1377965. doi: 10.3389/fmicb.2024.1377965 (PMC11018918; doi:10.3389/fmicb.2024.1377965)
Supplement: Supplementary file 1 [file Table_1.docx]

Table S1: The average nucleotide identity (ANI) values. 1) *B. sonorensis* NBRC 10123, 2) *P. polymyxa* DSM 36, 3) CamBx3, 4) *B. spizizenii* NBRC 101239, 5) *B. glycinifermentans* GO-13, 6) *B. haynesii* NRRL B-41327, 7) *B. cabrialesii* TE3, 8) *B. paralicheniformis* KJ-16, 9) *B. swezeyi* NRRL B-41294, 10) *B. inaquosorum* KCTC 13429, 11) *B. aerius* S00152.

| ANIb (%) | 1 | 2 | 3 | 4 | 5 | 6 | 7 | 8 | 9 | 10 | 11 |
| --- | --- | --- | --- | --- | --- | --- | --- | --- | --- | --- | --- |
| *B. sonorensis* NBRC 10123 | 100 | 70.1 | 81.3 | 74.2 | 85.4 | 81.5 | 74.4 | 81.4 | 83 | 74.5 | 72.2 |
| *P. polymyxa* DSM 36 | 72.1 | 100 | 72.3 | 71.7 | 71.7 | 72.2 | 72.1 | 72.2 | 71.1 | 72.2 | 72.4 |
| CamBx3 | 81.4 | 71 | 100 | 74.2 | 81 | 95.3 | 74.4 | 96.9 | 83.1 | 74.4 | 72.6 |
| *B. spizizenii* NBRC 101239 | 74.2 | 69.3 | 73.9 | 100 | 74 | 74.1 | 93.7 | 74 | 74.6 | 94.4 | 72.7 |
| *B. glycinifermentans* GO-13 | 85.4 | 69.7 | 80.9 | 74 | 100 | 80.9 | 74.1 | 81.2 | 82.2 | 74.2 | 72 |
| *B. haynesii* NRRL B-41327 | 81.4 | 69.7 | 95.1 | 74.2 | 81 | 100 | 74 | 95.1 | 83.4 | 74. | 72.2 |
| *B. cabrialesii* TE3 | 74.9 | 71.1 | 74.6 | 93.7 | 74.4 | 74.4 | 100 | 74.5 | 74.7 | 93.9 | 73.3 |
| *B. paralicheniformis* KJ-16 | 81.3 | 69.7 | 96.7 | 74.1 | 81.1 | 95.1 | 74 | 100 | 83 | 74 | 72.2 |
| *B. swezeyi* NRRL B-41294 | 83 | 69.8 | 83 | 74.7 | 82.2 | 83.5 | 74.4 | 83.1 | 100 | 74.5 | 72.4 |
| *B. inaquosorum* KCTC 13429 | 74.9 | 70.9 | 74.5 | 94.4 | 74.5 | 74.4 | 93.9 | 74.3 | 74.8 | 100 | 73.2 |
| *B. aerius* S00152 | 72.1 | 69.2 | 72.2 | 72.7 | 72 | 72.2 | 72.8 | 72.2 | 72.4 | 72.8 | 100 |


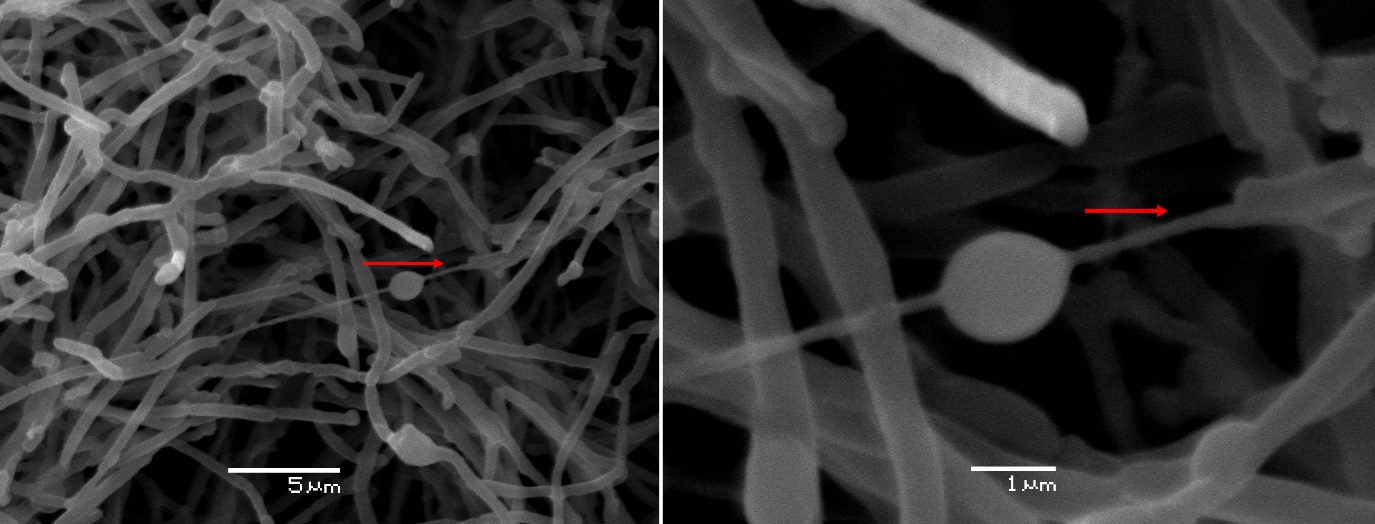


Fig S1. The SEM image of *B. paralicheniformis* CamBx3 (grown for 72 h) shows the presence of polysaccharides (marked with a red color arrow).
